# Cardiometabolic complications after Cushing’s disease remission

**DOI:** 10.1007/s40618-025-02572-x

**Published:** 2025-03-26

**Authors:** Irene Tizianel, Laura Lizzul, Alessandro Mondin, Giacomo Voltan, Pierluigi Mazzeo, Carla Scaroni, Mattia Barbot, Filippo Ceccato

**Affiliations:** 1 Department of Medicine-DIMED, University of Padova, Padova, Italy; 2 Endocrinology Unit, Department of Medicine DIMED, University Hospital of Padova, Via Ospedale Civile, 105, Padova, 35128 Italy

**Keywords:** Cushing’s disease, Hypercortisolism remission, Cushing’s disease medical treatment, Cushing’s disease medical therapy, Cushing’s disease neurosurgery

## Abstract

**Background and aim:**

Cushing’s disease (CD) is associated with phenotypic traits and comorbidities that may persist after the normalization of cortisol levels. Medical therapy is usually given in recurrent or persistent CD after transsphenoidal surgery. We aimed to investigate the impact of long-term normalization of daily cortisol secretion on clinical picture and cardiometabolic comorbidities, comparing surgical remission to medical treatment.

**Methods:**

Monocentric retrospective study, two- and five-years observation. Sixty CD patients, with sustained normal 24-h urinary free cortisol (UFC) levels, divided group 1 (surgical remission, *n* = 36) and group 2 (medical remission, *n* = 24).

**Results:**

Patients were different after achieving eucortisolism with surgery or medical treatment. Phenotypic traits: round face, dorsocervical fat pad, and bruisability persisted more prominently in the group 2, however abdominal obesity and muscle weakness persisted in both groups, especially in those patients with increased late-night salivary cortisol (LNSC). Hypertension: greater improvement was observed in group 1 (-31% vs. -5%, *p* = 0.04). Diabetes: less prevalent in group 1 after 2 years (2/36 vs. 9/24, *p* = 0.002), with a corresponding reduction in glucose-lowering treatments and persistence of impaired LNSC in diabetic patients (*p* < 0.001). Dyslipidemia: remained widespread in both groups, with minimal improvement over time (-22% in surgical and − 6% in medical cohort).

**Conclusions:**

Surgical remission leads to faster and sustained improvements in clinical phenotype. However, obesity, arterial hypertension, and dyslipidemia do not completely revert in five years, especially during medical treatment. Most comorbidities persist despite UFC normalization, due to impaired LNSC: the recovery of cortisol rhythms confirms the remission of hypercortisolism.

## Introduction

Cushing’s disease (CD) is caused by an adrenocorticotropic hormone (ACTH)-secreting pituitary tumor, resulting in persistent endogenous hypercortisolism. The cortisol excess leads to a typical clinical picture: round face, facial plethora, buffalo hump, cutaneous striae rubrae, easy bruising, proximal myopathy, weight gain with visceral obesity, hirsutism and acne [1–3]. Moreover, several comorbidities are cortisol-related: metabolic syndrome (visceral obesity, arterial hypertension, glucose intolerance or diabetes, and dyslipidemia), acquired thrombophilia, osteoporosis or vertebral fractures, immunological impairments with increased infection susceptibility, and psychiatric disorders [4]. The sum of physical changes and comorbidities leads to a reduced life expectancy and a worsening of the quality of life [5]. Pituitary trans-sphenoidal surgery (TSS) is the first-choice CD treatment [1]. Despite high remission rates (up to 90% in referral centers) [6], the risk of recurrence varies from 10 to 47% [7], especially in series with long-term follow-up. If surgery fails or is not feasible, cortisol excess can be managed with medical therapy. Not rarely, patients on cortisol-lowering therapy experience fluctuations of their cortisol levels, making outcome evaluations difficult and hardly standardized. The goals of CD treatment are to normalize cortisol levels, and to reduce the burden of comorbidities. The most used biochemical marker in clinical practice is urinary free cortisol (UFC), which estimates the cumulative daily secretion of cortisol, but does not offer information about cortisol rhythm [8].

In this study we compared two groups of CD patients with sustained normalization of 24-h UFC due either to post-surgical or medical cortisol-lowering therapy remission. The aim of the study was to analyze the impact of long-term normalization of hypercortisolism in terms of UFC, achieved with surgical or medical treatment, on endocrine parameters, cortisol-related clinical picture and comorbidities, in a five-years observation period of patients with CD.

## Materials and methods

### Subjects

Sixty CD patients were enrolled (75% female); the median age at diagnosis was 41 years (interquartile range [IQR] 32–52), followed at the Endocrinology Unit of Padua University Hospital from 2000 to 2021. This observational study was conducted in accordance with the STROBE (STrengthening the Reporting of OBservational studies in Epidemiology) guidelines [9]. The study, following the guidelines in the Declaration of Helsinki, was approved by the ethics committee of Padova University Hospital (PITACORA, protocol No. AOP3318, ethics committee registration 5938-AO-24), and all patients gave informed consent. All data are included in the Repository of the University of Padova [10].

**Fig. 1:**
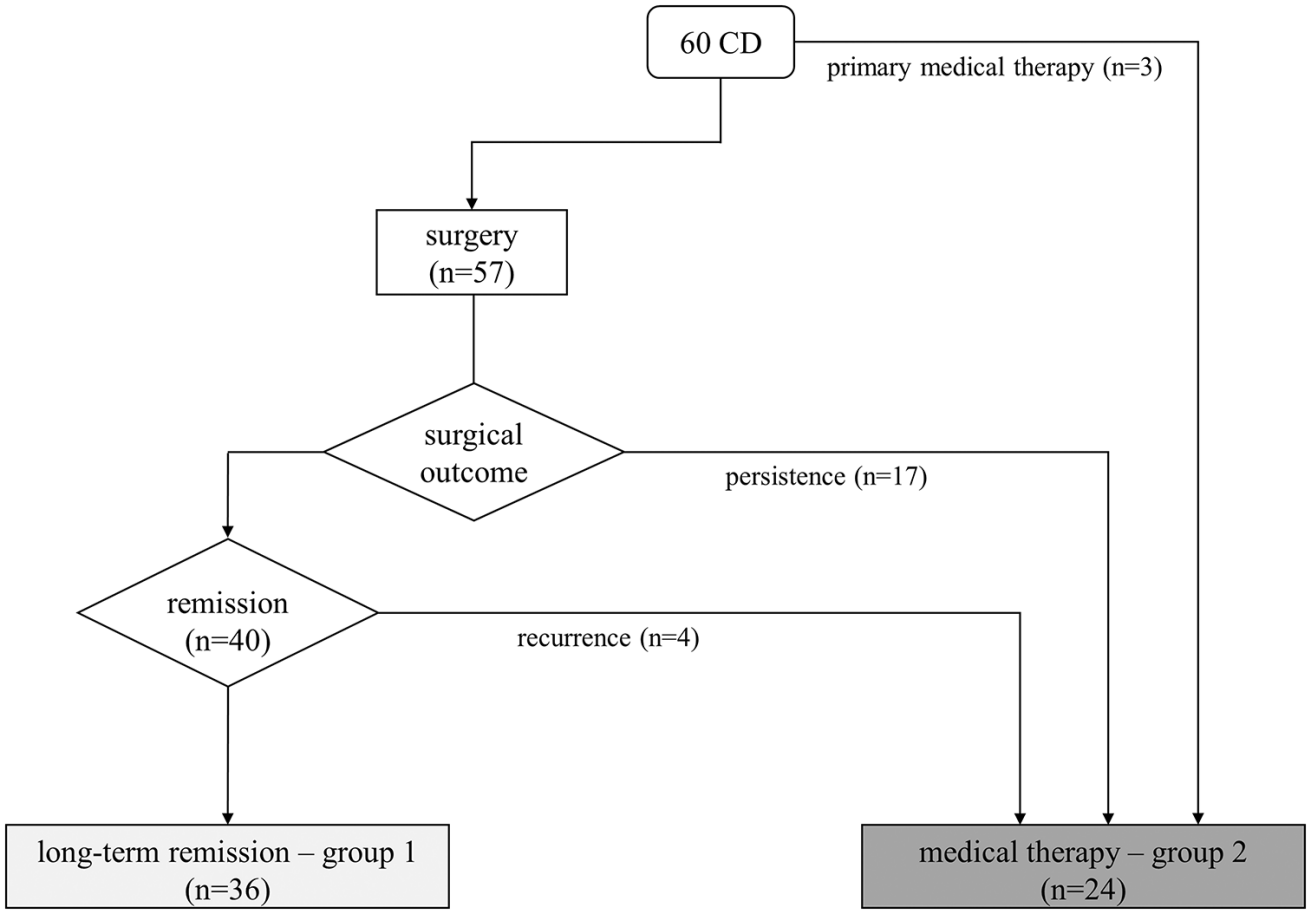
Treatment and outcome of the described cohort. Light gray box indicates those patients in group 1 (surgical remission, *n* = 36), dark gray box indicates the patients in group 2 that achieved normalization of UFC with medical therapy (*n* = 24, either primary or after surgical failure)

The first normalized UFC is considered as the starting point of observation at follow-up (two or five years). The cohort was divided into two cohorts: group 1 achieved CD remission after surgery, and group 2 achieved long-term eucortisolism during medical therapy. The inclusion criterion was 24-h UFC levels (mean of two collections) below the upper limit of normality during the observational period. Postoperative long-term adrenal insufficiency requiring substitutive glucocorticoid treatment (with hydrocortisone or cortisone acetate tablets) 12 months after surgery or new-onset hypopituitarism were considered exclusion criteria. The group 1 was made of 36 patients (69% female) in remission after successful TSS. The second group consisted of 24 patients (83% female) on long-term medical treatment for CD persistence (*n* = 17) or relapse (*n* = 4) after surgery and three patients in primary medical therapy for poor surgical eligibility, as shown in Fig. 1. Within group 2, nine patients underwent previous radiotherapy without efficacy, at least 5 years before reaching adequate biochemical control with medical treatment; none developed hypopituitarism. 14/24 patients (58%) were treated with a monotherapy and 11/24 (46%) with combined therapies during the observation period. Details on medical therapies are shown in Table 1. In particular, 3 patients were treated with metyrapone + pasireotide s.c., 1 with metyrapone + ketoconazole, 2 with ketoconazole and cabergoline, 1 with metyrapone + cabergoline, 1 with metyrapone + ketoconazole + cabergoline, 1 with metyrapone + ketoconazole + pasireotide s.c., 1 with metyrapone + ketoconazole + pasireotide s.c. + cabergoline. Metyrapone and ketoconazole were administered two/three times a day, pasireotide s.c. twice daily and cabergoline once daily in the evening.

**Table 1:** Cortisol-lowering drugs, dose, and time in treatment of subjects treated with a single and combined lines of therapy

Drug	Monotherapy						Combination Therapy					
	Number of patients treated		Dose		Time in treatment (months)		Number of patients treated		Dose		Time in treatment (months)	
	2 aa (*N* = 14)	5 aa (*N* = 8)	Mean	Range	Mean	Range	2 aa (*N* = 10)	5 aa (*N* = 3)	Mean	Range	Mean	Range
Pasireotide µg	5	4	750 (µg/die)	(300–1200)	44	(24–60)	5	2	750 (µg/die)	(300–1200)	42	(24–60)
Cabergoline mg	0	0					5	2	2.3 (mg/week)	(1.5–3)	19.2	(8–26)
Ketoconazole mg	3	3	366 (mg/die)	(200–600)	32	(16–56)	6	3	370.8 mg/die	(200–600)	18.8	(7–26)
Metyrapone mg	2	0	666 (mg/die)	(250–1250)	25.3	(21–24)	8	3	661.25 mg/die	(250–1000)	26.25	(15–38)
Osilodrostat mg	4	1	5.9 (mg/die)	(1–14)	29	(22–44)	0	0				

All 60 patients completed at least 2 years of follow-up; a long-term 5-years evaluation was available in 43 patients of the original cohort (32 after surgery and 11 with medical therapy). Baseline characteristics of the two cohorts are reported in Table 2.

**Table 2:** Baseline characteristics of the two groups and previous treatment modalities

	Group 1 (surgical remission) *n* = 36	Group 2 (medical remission) *n* = 24
Sex M (%F)	25/11 (69%)	20/4 (83%)
BMI (kg/m2)	25 (7)	27 (± 6)
Age at CD diagnosis (years)	41 (35.7–49.5)	41.5 (36.8–48.9)
Exposure time to excess glucocorticoids (years)	1 (± 1)	12.5 (± 9)^a^
Age at remission/UFC normalization (years)	42.5 (± 19)	49.5 (± 13)
Number of surgeries	36§	21/24 (87%)*
Radiotherapy	0	8/24 (33%)

Data are presented as medians and interquartile range (in brackets) or absolute numbers and percentages

^a^*p <* 0.001

*14 (one TNSN) + 7 (two TNSN)

^§^5 reintervention within the group

## Data collection and study design

Two researchers retrieved clinical and biochemical data independently from the local digital medical records. We considered as baseline visit the clinical and endocrine evaluation performed with active hypercortisolism. Therefore, the baseline visit consists in the pre-surgical evaluation in group 1, and in the post-surgical confirmation of active hypercortisolism in those in medical treatment (or diagnosis in case of primary treatment, group 2).

We considered clinical and biochemical outcomes during routine follow-up at two- and five-years in each group, starting from surgical remission or the beginning of a stable normalization of UFC under medical therapy. CD diagnosis was based on at least two parameters among 24-h UFC above the upper normal limit (ULN, at least two collections), unsuppressed cortisol levels (> 50 nmol/L) after 1 mg overnight dexamethasone test (1 mg-DST) or late-night salivary cortisol (LNSC) > ULN (at least two samples). In all subjects, CD diagnosis was considered in case of normal-high ACTH levels, positive response to dynamic tests (corticotropin-releasing hormone or desmopressin test, high-dose dexamethasone test), and, two cases, with petrosal sinus sampling (BIPSS) [11]. Long-term remission after TSS was defined through normal UFC, combined with serum cortisol levels < 50 nmol/L in the first month after surgery and need of glucocorticoid replacement therapy. A relapse of CD was defined as the reappearance of the typical signs and symptoms of CD associated with the alteration of at least two first-line screening tests. Presence/absence of clinical signs of CD (round face, facial rubor, buffalo hump, bruising, cutaneous red striae, acne, hirsutism and oligo/amenorrhea in females) were evaluated during outpatient visits by expert endocrinologists. The presence of hirsutism in females was measured according to the Ferriman–Gallwey score > 8 (extent of hair growth in 9 locations was rated 0–4). Proximal muscle strength was diagnosed if patients were not able to stand up from a low seated position with anteriorly extended arms. Bodyweight, body mass index (BMI), waist and hip circumference, systolic (SBP), and diastolic blood pressure (DBP) were assessed with calibrated tools. Overweight was diagnosed in patients with BMI 25–30 kg/m^2^, obesity with BMI > 30 kg/m^2^. Visceral obesity was diagnosed as waist circumference ≥ 94 cm in men and ≥ 80 cm in women, or with a waist/hip ratio (WHR) ≥ 1 according to International Diabetes Federation criteria. Arterial hypertension was diagnosed for SBP above 140 mm Hg and/or DBP above 90 mm Hg and/or in patients on antihypertensive drugs. Diabetes mellitus (DM) was diagnosed according to American Diabetes Association criteria or when patients were taking antidiabetic medication. Dyslipidemia was diagnosed when low-density lipoprotein (LDL) calculated cholesterol was ≥ 100 mg/dL and hypertriglyceridemia when triglycerides were ≥ 150 mg/dL or when patients were on lipid-lowering medication. The presence of carotid vascular disease (CVD) has been assessed by supra-aortic vessels duplex ultrasound. Cushing’s cardiomyopathy (CCM) was diagnosed by doppler echocardiography with evidence of impaired relaxation and left ventricular filling pattern. The medical history was checked for cardiovascular disease (acute coronary syndrome, ACS) in all cases. A shortened activated partial thromboplastin time (aPTT < 29 s) defined pro-thrombotic status.

## Assays

All biochemical analyses were carried out in an ISO15189:2012-accredited clinical laboratory [12], cortisol levels have been measured in urine or saliva with a mass-spectrometry home-made validated method. UFC was determined by a home-brew liquid chromatography-mass spectrometry (LC-MS/MS) method (intra-assay/interassay coefficient of variation [CV] < 6%/< 8%) since 2011 [13], previously by a radio-immunometric assay (Radim, intra-assay/interassay CV < 3%/< 9%). The patients were instructed to discard the first morning urine void and to collect all urine for the next 24 h, so that the morning urine void on the second day was the final collection. The sample was kept refrigerated from collection time until it was analyzed: normal range for UFC is 16–168 nmol/24 h.

Salivary cortisol was measured by a radio-immunometric assay (Radim, intra-assay/interassay CV < 3%/< 9%) until 2014 [14], after then by LC-MS/MS method (intra-assay/interassay CV < 6%/< 8% [15]). In order to prevent food or blood contamination, samples were collected at least 30 min after subjects had eaten, brushed their teeth, smoked or assumed liquorice; undertaken using Salivette^®^ devices containing a cotton swab with or without citric acid (Sarstedt, Nümbrecht, Germany). The sample was stored at − 80 °C, before analyses [15].

The 1-mg DST test was performed orally assuming 1 mg of dexamethasone between 11 P.M. and midnight, sampling serum cortisol the next morning at 8 A.M. Serum dexamethasone levels, routinely evaluated since 2017, were adequate in all cases [16]. Serum cortisol (RRID: AB_2810257) and ACTH (RRID: AB_2783635) were determined by immune-chemiluminescence assay (Immulite 2000, Siemens Healthcare). Dynamic second-line tests and BIPSS were performed according to international standards.

### Statistical analysis

Data were analyzed using SPSS Software for Windows, version 24.0 (SPSS Inc). Data are reported as medians and interquartile range or as percentages. The comparison between continuous variables was performed by non-parametric Wilcoxon test or Mann–Whitney test, as appropriate. The comparison between categorical variables was performed by the χ2 test. The correlation between continuous variables was performed by linear regression analysis. The level of significance for the overall difference between the groups was tested with one-way ANOVA. A p value < 0.05 was considered statistically significant.

## Results

### Endocrine evaluation

At baseline the two groups were similar for morning serum/salivary cortisol, LNSC, cortisol after 1 mg DST and morning ACTH levels (Table 3); UFC levels were higher in the surgical cohort (*p* < 0.001). Endocrine parameters were not influenced by sex and BMI. At baseline, all patients had impaired salivary cortisol rhythm with increased LNSC and inadequate cortisol suppression after 1-mg DST. At two years the recovery of salivary cortisol rhythm was observed in 97% of patients after surgery and 50% of patients during medical therapy. The only patient who did not show recovery of cortisol rhythm in the surgical cohort had LNSC of 5.4 nmol/L (range 0.5–2.6 nmol/L), with adequate cortisol suppression after 1-mg DST and sustained normal UFC: it was considered a false-positive due to residual minor depression state.

**Table 3:** Biochemical pattern at baseline and during the follow-up

Biochemical parameter		Group 1 (surgical remission)	Group 2 (medical remission)	*P*
UFC (nmol/24 h, range 16–168)	Baseline	1158 (680–2180)	277 (217.5–1152)	*p* < 0.001
	2 years	65 (46–93)	86 (55.5–130)	*p* = 0.02
	5 years	68 (38–101)	81 (46.5–125)	n.s.
Morning serum cortisol (nmol/L)	Baseline	588 (517.5–676.5)	563 (364–654)	n.s.
	2 years	279 (227.5–356)	384 (262–464)	*p =* 0.006
	5 years	309 (248.5–344.5)	419 (268–502)	*p =* 0.005
Morning salivary cortisol (nmol/L)	Baseline	21.5 (8.1–25.8)	15.6 (7.9–20.2)	n.s.
	2 years	5.9 (4.2–10)	12.2 (7.7–16.3)	*p =* 0.006
	5 years	4.7 (3.7–9.3)	7.5 (3.3–9.5)	n.s.
Late-night salivary cortisol (nmol/L, range 0.5–2.6)	Baseline	12.7 (6.2–16)	8.9 (6.6–12.9)	n.s.
	2 years	0.9 (0.5–1.2)	3.2 (1.7–4.9)	*p <* 0.001
	5 years	1.1 (0.7–1.5)	2.3 (1.9–4.7)	n.s.
Cortisol post 1-mg DST (nmol/L)	Baseline	386 (215–519)	244	n.s.
	2 years	27 (21–39)	70 (55–149)	*p <* 0.001
	5 years	35 (19–44)	75 (60–130)	*p <* 0.001
ACTH (ng/L)	Baseline	47 (38.5–65)	44 (30.5–65)	n.s.
	2 years	19 (14–30)	39 (20.9–81.5)	*p =* 0.05
	5 years	20 (16–33.5)	42 (18.8–83.5)	*p =* 0.02

*p* at 2y and 5y refers to the baseline. n.s.: non-significative. Data are expressed as medians and interquartile range in brackets

Adequate cortisol suppression after 1-mg DST (both with normal UFC and LNSC) was observed in 34 out of 36 patients (94%) in the surgical cohort; the two patients who did not show complete cortisol suppression after 1-mg DST had cortisol levels of 60 and 119 nmol/l, respectively. On the contrary, as per selection criteria, none of the patients in group 2 presented suppressed cortisol after 1-mg DST.

At 5 years follow-up, all cases in the surgical cohort had suppressed cortisol after 1-mg DST and normal salivary cortisol rhythm, whereas in group 2 9% had suppressed cortisol after 1-mg DST and 36% recovered salivary cortisol rhythm. At 5 years, UFC and salivary cortisol levels (either morning or late night) were similar in the two groups, while the median value of serum cortisol after 1-mg DST remained not adequately suppressed (median 75 nmol/L, from 18 to 257 nmol/L) during medical therapy (See Table 3). In group 2, patients on combined therapy had higher UFC (102 vs. 76 nmol/24h *p =* 0.03) and LNSC (2.4 vs. 1.9 *p =* 0.05) at 5 years, compared to patients on monotherapy.

**Fig. 2:**
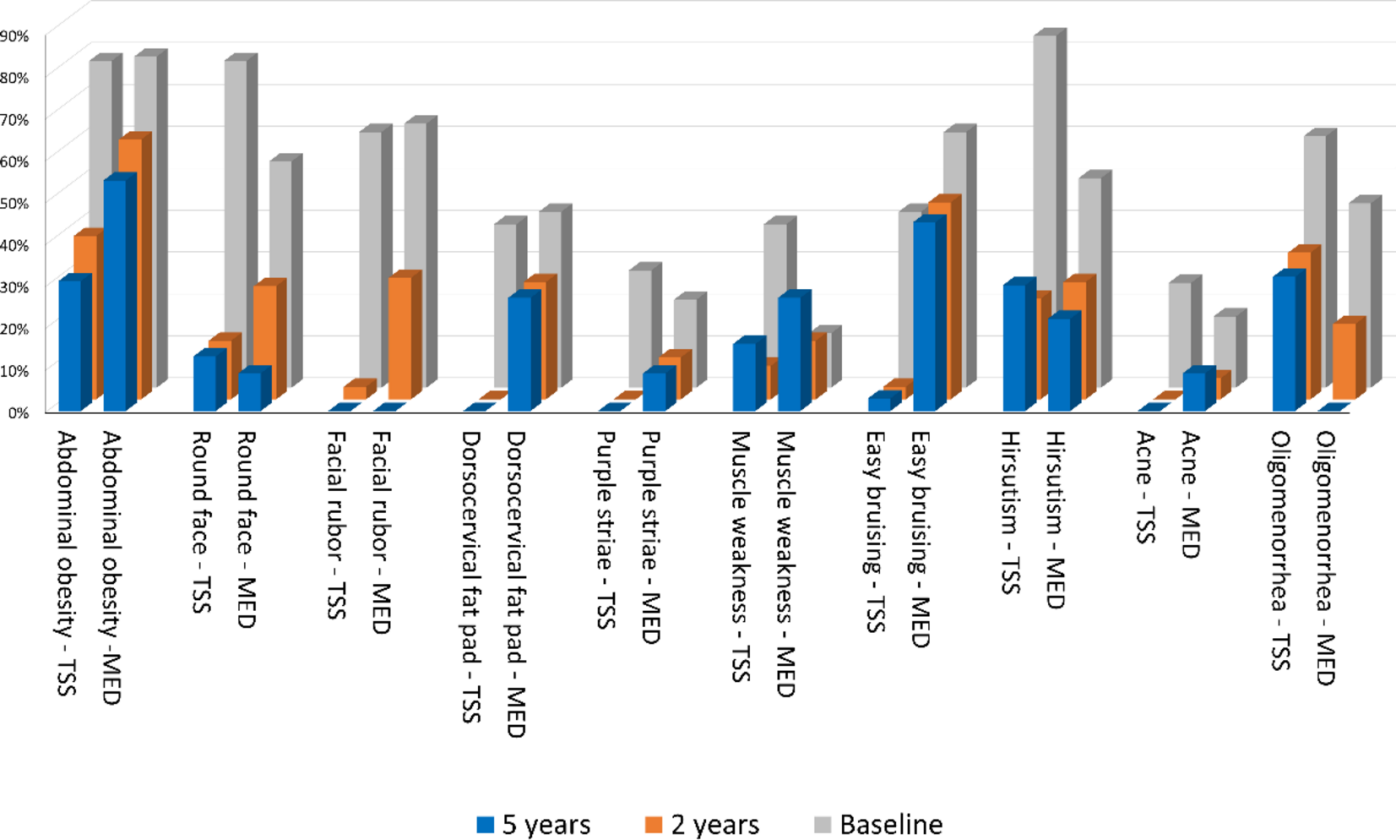
Signs and symptoms of hypercortisolism at baseline (grey bars), two-years (orange bars) and five-years (blue bars) follow up after surgical (TSS) or medical remission (MED)

Hirsutism, abdominal obesity, round face and facial rubor were prevalent in group 1 at baseline. On the contrary, the abdominal obesity, facial rubor and easy bruising were most commonly found in the medical cohort. The prevalence of facial rubor, buffalo hump and bruisability was higher after medical than surgical remission after 2 years of eucortisolism; at 5 years the prevalence of buffalo hump and bruisability was higher in patients under drug therapy as well (Table 4; Fig. 2). Higher levels of UFC at baseline were observed in all patients with proximal myopathy (*p* < 0.001).

**Table 4:** Two- and five-years changes in clinical phenotype from baseline in group 1 and group 2

		Group 1 (surgical remission)	Group 2 (medical remission)	*P*
Weight (kg)	Baseline	68	66	n.s.
		(49–120)	(56–89)	
	2 years	64	66	
		(51–115)	(56–89)	
	Δ from baseline	−6%	=	
	(%)			
	5 years	65	66	
		(50–120)	(56–89)	
	Δ from baseline (%)	−4%	=	
BMI (kg/m^2^)	Baseline	25	28	n.s.
		(19–40)	(23–30)	
	2 years	24	27	
		(19–38)	(20–32)	
	Δ from baseline	−4%	−3%	
	(%)			
	5 years	24	28	
		(19–40)	(23–33)	
	Δ from baseline		=	
	(%)	=		
Waist-Hip Ratio (cm)	Baseline	0.94	1	n.s.
		(0.8–1.1)	(0.9–1.1)	
	2 years	0.94	0.95	
		(0.8–1.1)	(0.9–1.1)	
	Δ from baseline	=	=	
	(%)			
	5 years	0.94 (0.7–1.1)	0.94	
			(0.9–1.4)	
	Δ from baseline	=	=	
	(%)			
Waist (cm)	Baseline	91.5 (68–118)	99	*p =* 0.04 at 2y
			(85–112)	
	2 years	89.5	97 (78–110)	
		(67–115)		
	Δ from baseline	−2%	−2%	
	(%)			
	5 years	88.5 (63–112)	94	
			(78–124)	
	Δ from baseline	3%	−5%	
	(%)			
Abdominal obesity	Baseline	28/36	19/24	n.s.
		(78%)	(79%)	
	2 years	14/36	14/24	
		(39%)	(58%)	
	Δ from baseline	−50%	−22%	
	(%)			
	5 years	10/32	6/11 (54.5%)	
		(31%)		
	Δ from baseline	−60%	−31%	
	(%)			
Round face	Baseline	28/36	13/24	n.s.
		(78%)	(54%)	
	2 years	5/36	6/24	
		(14%)	(25%)	
	Δ from baseline	−82%	−50%	
	(%)			
	5 years	4/32	1/11	
		(12%)	(9%)	
	Δ from baseline	−84%	−78%	
	(%)			
Facial rubor	Baseline	22/36	15/24	*p =* 0.008 at 2y
		(61%)	(62%)	
	2 years	1/36	6/24	
		(3%)	(2%)	
	Δ from baseline	−95%	−53%	
	(%)			
	5 years	0/32	0/11	
	Δ from baseline	−100%	− 100%	
	(%)			
Dorsocervical fat pad	Baseline	14/36	10/24	*p =* 0.001 a 2 y e *p* = 0.04 a 5y
		(39%)	(42%)	
	2 years	0/36	6/24	
			(25%)	
	Δ from baseline	−100%	−33%	
	(%)			
	5 years	0/32	3/11	
			(27%)	
	Δ from baseline	=	−36%	
	(%)			
Purple striae	Baseline	10/36	5/24	n.s.
		(28%)	(21%)	
	2 years	0/36	2/24	
			(8%)	
	Δ from baseline	−100%	−55%	
	(%)			
	5 years	0/32	1/11	
			(9%)	
	Δ from baseline	=	−57%	
	(%)			
Muscle weakness	Baseline	14/36	3/24	*p =* 0.04 baseline
		(39%)	(12%)	
	2 years	3/36	4/24	
		(8%)	(16%)	
	Δ from baseline	−79%	=	
	(%)			
	5 years	2/32	3/11	
		(16%)	(27%)	
	Δ from baseline	−59%	=	
	(%)			
Easy bruising	Baseline	15/36	14/24	*p <* 0,01 at 2 and 5 y
		(42%)	(58%)	
	2 years	1/36	11/24	
		(3%)	(45%)	
	Δ from baseline	−93%	−22%	
	(%)			
	5 years	1/32	5/11	
		(3%)	(45%)	
	Δ from baseline	=	=	
	(%)			
Hirsutism	Baseline	21/25	10/20	*p =* 0.02 baseline
		(84%)	(50%)	
	2 years	6/25	6/24 (25%)	
		(24%)		
	Δ from baseline	−71%	−44%	
	(%)			
	5 years	7/23	2/9	
		(30%)	(22%)	
	Δ from baseline	−64%	−56%	
	(%)			
Acne	Baseline	9/36	4/24	n.s.
		(25%)	(17%)	
	2 years	0/36	1/24	
			(3%)	
	Δ from baseline	−100%	−71%	
	(%)			
	5 years	0/32	1/11	
			(9%)	
	Δ from baseline	=		
	(%)		−9%	
Oligomenorrhea	Baseline	12/20	7/16	n.s.
		(60%)	(44%)	
	2 years	7/20	4/24	
		(35%)	(17%)	
	Δ from baseline	−42%	−59%	
	(%)			
	5 years	6/19	0/5	
		(32%)		
	Δ from baseline	−68%	=	
	(%)			

*p* at 2y and 5y refers to the baseline. n.s.: non-significative, p value is specified only when significant. Data are presented as medians and interquartile range (in brackets) or absolute numbers and percentages

## Arterial hypertension

Arterial hypertension (AH) was the most frequent comorbidity in both groups at baseline, with similar distribution in the two groups (Table 5). The prevalence of AH decreased after two years in both groups, especially in the surgical cohort (64% vs. 44% in group 2, *p <* 0.001; 75% vs. 71% *p =* 0.003), with no further improvement after five years. Overall, hypertensive patients were older at diagnosis (45yrs vs. 31y; *p <* 0.001) and with larger BMI (29 vs. 25 kg/m^2^; *p =* 0.03). Median UFC, morning salivary cortisol and LNSC, and 1-mg DST were not different in patients with/without AH at baseline and at 2 years. SBP and DBP values were similar in the two cohorts and were not correlated to UFC, LNSC or 1-mg DST throughout the follow-up. At 2 years, hypertensive patients had higher levels of morning salivary cortisol and LNSC with impaired rhythm (respectively 10.4 vs. 6 nmol/L, *p =* 0.01 and 3.2 vs. 1 nmol/l, *p =* 0.007). SBP and DBP values did not change during the five-years observation time in both groups; however, the number of anti-hypertensive drugs was higher in group 2 than in group 1 (*p* = 0.007). Overall patients treated with metyrapone showed higher values of DBP at 2 years (mean 89.4 vs. 81.7 mmHg, *p =* 0.01), the prevalence of AH did not differ from patients with other medical treatments.

**Table 5:** Two- and five-years changes in cardio-metabolic cortisol-related comorbidities of CD from baseline in group 1 and group 2

		Group 1 (surgical remission)	Group 2 (medical remission)	*P*
Hypertension	Baseline	23/36	18/24	p = 0.04 2y
		(64%)	(75%)	
	2 years	16/36	17/24	
		(44%)	(71%)	
	Δ from baseline (%)	−31%^*^	−5%^#^	
	5 years	16/32	08/11	
		(50%)	(73%)	
	Δ from baseline (%)	=	−3%	
SBP	Baseline	140	135	n.s.
		(105–190)	(120–160)	
	2 years	130	130	
		(100–145)	(110–145)	
	Δ from baseline (%)	−7%	−4%	
	5 years	130	122.5	
		(100–150)	(100–160)	
	Δ from baseline (%)		−9%	
		=		
DBP	Baseline	90	85	n.s.
		(70–120)	(70–90)	
	2 years	80	82.5	
		(60–100)	(80–100)	
	Δ from baseline (%)	−11%	−3%	
	5 years	85 (60–100)	80	
			(60–90)	
	Δ from baseline (%)	−5%	−6%	
N° Drugs for hypertension	Baseline	1.14	1.2	p = 0.007 a 2y
		(± 1,3)	(± 1,1)	
	2 years	0.59	1.2	
		(± 0.8)	(± 1)	
	Δ from baseline (%)			
	5 years	0.66	0.8	
		(± 0.8)	(± 0.8)	
	Δ from baseline (%)			
Diabetes	Baseline	28/36	19/24	n.s
		(78%)	(79%)	
	2 years	14/36	5/14	
		(39%)	−36%	
	Δ from baseline (%)	−50%	−22%	
	5 years	11/32	1/5 (20%)	
		(31%)		
	Δ from baseline (%)	−60%	−31%	
HbA1c	Baseline	37	40	n.s.
		(35–44)	(32–43)	
	2 years	37	36	
		(33–43)	(33–40)	
	Δ from baseline (%)	=	−8%	
	5 years	37	36	
		(35–43)	(34–40)	
	Δ from baseline (%)	=	=	
N° glucose lowering drugs	Baseline	0.1	0.2	p = 0.01 baseline
		(± 0.3)	(± 0.4)	p = 0.001 2y
	2 years	0	0.3	p = 0.02 5y
			(± 0.7)	
	Δ from baseline (%)			
	5 years	0	0.2	
			(± 0.7)	
	Δ from baseline (%)			
***N° insulin treated patients***	Baseline	3/36 (8%)	2/24	n.s.
	2 years	0/36	0/14	
	Δ from baseline (%)			
	5 years	0/32	1/4 (20%)	
	Δ from baseline (%)			
Dyslipidemia	Baseline	19/36	15/24	p = 0.01 5y
		(54%)	(62%)	
	2 years	15/36	14/24	
		(42%)	(58%)	
	Δ from baseline (%)	−22%	−6%	
	5 years	15/32	10/11	
		(47%)	(91%)	
	Δ from baseline (%)	=	=	
LDL cholesterol > 100 mg/dL	Baseline	14/36	3/24	n.s.
		(39%)	(12.5%)	
	2 years	11/36	4/24	
		(8%)	(16%)	
	Δ from baseline	−79%	=	
	(%)			
	5 years	11/32	3/11	
		(16%)	(27%)	
	Δ from baseline (%)	−59%	=	
Triglycerides mmol/L	Baseline	1.14	1.14	n.s.
		(0.69–3.24)	(0.55–3.64)	
	2 years	1.12	1.4	
		(0.39–2.73)	(0.94–2.63)	
	Δ from baseline (%)	−2%	22%	
	5 years	0.97	1.38	
		(0.45–2.52)	(0.56–3.6)	
	Δ from baseline (%)	−15%	21%	

*p* at 2y and 5y refers to the baseline. n.s.: non-significative, p values are specified only when significant. Data are presented as medians and interquartile range or mean and standard deviation (in brackets) or absolute numbers and percentages. SBP: systolic blood pressure, DBP: diastolic blood pressure. * *p <* 0.001, # *p =* 0.003

## Glucose metabolism

DM prevalence at baseline did not show a correlation with BMI and age at CD diagnosis. DM prevalence was similar in group 1 and 2 after two and five years of follow-up. The follow-up analysis of DM was performed excluding patients in pasireotide, since its known impact in glucose metabolism. In both groups, median UFC, morning salivary and LNSC, and 1-mg DST were similar in patients with/without DM at baseline. At 5 years, patients with diabetes had higher levels of morning salivary cortisol and LNSC with impaired cortisol rhythm (respectively 15 vs. 7 nmol/L, *p <* 0.001 and 5.4 vs. 1.5 nmol/l, *p <* 0.001). None of the explored hormonal parameters was correlated with HbA1c levels in both groups at any time point considered. The number of antidiabetic drugs was higher after medical than surgical remission (Table 5).

As expected, patients treated with pasireotide had higher incidence of newly onset DM at 2- and 5 years (*p =* 0.02 and *p =* 0.05 respectively) and required more antidiabetic drugs at 2- and 5 years (*p =* 0.002, *p =* 0.05) or insulin units at 5 years (*p =* 0.03). HbA1c levels during pasireotide were higher than patients treated with other drugs (55.6 vs. 38 nmol/l, *p =* 0.002), requiring a higher number of antidiabetic drugs (*p =* 0.008). Patients on combined therapy with pasireotide had higher rates of DM at 2- and 5 years (*p <* 0.001 and *p =* 0.01) and used more antidiabetic drugs at 2- and 5 years (*p =* 0.004, *p =* 0.01) than those on monotherapy.

### Lipid metabolism

The prevalence of dyslipidemia was similar in the two groups at baseline and after two years, and higher in the medical remission cohort after five years (*p =* 0.01). Overall, dyslipidemic patients were older at diagnosis (46y vs. 36y; *p =* 0.006) and had higher BMI (30 vs. 25 kg/m^2^; *p <* 0.001). There was no correlation between hormone parameters and LDL or triglycerides levels. Lipid profile was similar between patients treated with different drugs.

### Vascular disease and coagulative profile

There was no difference between the two groups, at baseline, in the prevalence of carotid vascular disease, history of ACS, and CCM; at 5 years, in both groups, no patient had a worsening of a previously diagnosed stenosis, or novel diagnosis of CVD, ACS and CCM.

The median aPTT value at baseline was in the pro-thrombotic range in both groups (25s), without sex and BMI differences. No correlation was observed between aPTT and UFC, LNSC and 1-mg DST levels. Patients who manifested easy bruising, had shorter aPTT at 2- and 5 years (median 24 vs. 27s, *p* = 0.03). aPTT does not increase within both groups at 2- and 5-years and aPTT was shorter during medical therapy compared to surgical remission both after 2 and 5 years (22.5s vs. 27s, *p* = 0.02 at 2y and 23.5s vs. 27.9s, *p* = 0.02 at 5y).

## Discussion

The impact of CD remission on clinical picture and hypercortisolism-related comorbidities is still controversial. The current knowledge suggests that long-term CD surgical remission is associated with increased metabolic and vascular damage, not only if compared to active disease, but also even after long-term normalization of cortisol secretion [17]. If CD recurs after successful TSS, or if surgery fails/is not feasible, cortisol excess can be treated with medical therapy. Likewise, long-term studies (> 2 years) on the clinical effects of medical therapy on CD are lacking. Some prospective registry studies have been published [1], only one retrospective study on long-term use of ketoconazole described a multicentric cohort of CD patients without a control group [18].

In our study, we enrolled 60 patients with CD diagnosed and treated in a single tertiary care center, with sustained and long-term (2 and 5 years) UFC normalization after surgery or during medical therapy. As expected, UFC levels at baseline were different in the two groups, due to the distinct starting point of medical history: a patient with persistent-recurrent CD after pituitary surgery presents with lower UFC than the new diagnosis. After surgical remission, patients achieved the recovery of salivary cortisol rhythm and the complete suppression of cortisol after 1-mg DST (investigated after substitutive glucocorticoid treatment discontinuation) in almost all cases. On the contrary, if eucortisolism is achieved with long-term medical therapy the recovery of salivary cortisol rhythm was observed only in half of patients and only few of them showed cortisol suppression after 1-mg DST within the 5 years observation time. Patients who were more resistant to the recovery of cortisol rhythm were more likely to receive combined treatment, even if no treatment is superior to others in normalizing salivary cortisol rhythm, in line with previous reports [1, 18, 19].

Within 2 years, patients in the surgical remission group showed a marked improvement of all phenotypic traits common at CD diagnosis compared to those in medical therapy. As observed also in other series of CD patients in remission [20], abdominal obesity persisted more than other clinical features over time, leading to an impaired body composition especially in the medically treated group [21]. Considering hyperandrogenism, acne improvement was more relevant at 2 and 5-years of follow up, probably due to a differential effect of ACTH-dependent adrenal androgens compared to hirsutism.

The impaired cortisol rhythm was a predictor of the long-lasting of most CD phenotypic features, as round face, buffalo hump, facial rubor, abdominal obesity, proximal myopathy and bruisability. A more severe clinical phenotype at baseline can explain a reduced control of hypercortisolism in monotherapy, requiring drug combination, and signs or symptoms are likely to persist despite the normalization of UFC [22]. In this study, no medication outperformed the others in terms of recovery from the CD phenotype.

The aetiology of hypertension and dyslipidemia is known to be heterogeneous, since both are influenced also by age at diagnosis and BMI, causing low rates of remission after UFC normalization [23, 24]. Arterial hypertension showed a decreasing trend with the best response within 2 years after UFC normalization only after surgical remission. Patients with disrupted salivary cortisol rhythm were more likely to remain hypertensive during the 5 years follow-up. Likewise, DM persistence during follow up correlates to impaired salivary cortisol rhythm and not with UFC. This finding is in contrast with the observations of Schernthaner-Reiter et al. [25]. on CD remission, and, on the contrary, supports data described by Guarnotta et al. [22]. Newell-Price et al.. recently found that when UFC and LSNC are both normal in patients treated with pasireotide, the rise in HbA1c levels is less evident than in patients with normal UFC but uncontrolled LNSC [26]. This observation underlines the importance of the impaired cortisol rhythm in the glucose impairment pathogenesis in CD. During the 5 years observation time, a worsening of previously diagnosed cardiovascular conditions, or novel acute vascular events, was not observed in both groups. This finding suggested that normalized UFC and intensive treatment of cardio-metabolic CD comorbidities play a fundamental role in reducing cardiovascular mortality [27]. A minor impact of CD therapy was observed in dyslipidemia, which persisted in both groups, with minimal improvement over time (−22% in surgical and − 6% in medical cohort). The criterion of 100 mg/dL LDL cut-off identifies a moderate CV risk reflecting the main focus of the study: the assessment of cardiometabolic complication after CD remission, assuming that they present a lower cardiovascular risk compared to patients with overt hypercortisolism.

Plasma hypercoagulability, with shortened aPTT, was found in all patients with active hypercortisolism. In the 5 years observation time, this parameter showed latency in increasing in both groups and in none achieved normality (> 28s). As previously observed in other studies, no correlation is observed between aPTT and any of the explored hormonal parameters [22, 28]. At 2- and 5 years, instead, shorter aPTT was observed during medical treatment than after surgical remission cohort. In both groups a shorter aPTT was associated with bruisability, which is related to impaired LNSC, strengthening the role of the impaired cortisol rhythm as a major driver of hypercoagulability. Also, Ferrante et al.. observed the long latency of plasma hypercoagulability, persisting for years after biochemical remission of CD: in that series thrombophilia appeared to be reversible within 5 years [29], while in our cohort the recovery takes longer.

Additionally, sexual differences characterize patients with patients with Cushing’s syndrome and hypogonadism in hypercortisolism is known to further increase the cardiovascular risk [30, 31]. However, it was not an interfering factor in our study population since hypopituitarism was considered an exclusion criterion, no case of new-onset hypogonadism was reported (even in male patients treated with ketoconazole), and the menopause transition in six women during the observation was not considered relevant.

The limits of the present study are its retrospective design, the variability of concomitant treatments, the heterogenous combinations of medical therapy used in clinical practice, the presence of treatment-specific adverse events that mimic the effects of hypercortisolism (such as pasireotide-induced DM and hypertension with metyrapone), the unpredictable effect of previous treatments, including radiotherapy. We considered UFC and LNSC as markers of hypercortisolism remission; nonetheless we acknowledge that both of them present some limitations, especially during medical treatment. The former considers the whole cortisol secretion during the day, and albeit UFC normalization is the main outcome of all trials for medical treatment [32, 33] it does not detect mild hypercortisolism. On the other hand, a normal LNSC does not fully reflect a normal circadian rhythm: only high cortisol levels in the morning with a decline in the night are able to restore clock-related activities [34]. 

Its strengths are the complete patient characterization in a single tertiary care center, the comparative study design, and the standardized protocols for diagnosis and long-term follow-up. In particular, samples have been processed within a single laboratory with accurate methods (LC-MS for urinary and salivary steroids), and all endocrine aspects of hypercortisolism were considered (overall daily cortisol production by UFC, circadian cortisol rhythm, and the recovery of the hypothalamic-pituitary axis by 1-mg DST overnight test).

To conclude, despite UFC normalization in both groups during follow-up, surgical remission results in more rapid and relevant improvements in CD phenotype and comorbidities. During medical therapy the UFC levels can be higher than after surgery, although in the normal range, and the normalization of LNSC is not always achieved: both conditions suggests that stricter criteria should be considered to define eucortisolism in patients with CD under medical treatment. Conditions such as obesity, hypertension, dyslipidemia, and hypercoagulability are not completely reversible in a 5-year observation time even in the surgical remission group. This observation underlines that all the comorbidities, independently of the normalization of UFC, must be intensively treated. Moreover, UFC normalization should not be considered the only biochemical goal to be reached, since the persistence of comorbidities seems to be more related to an impaired cortisol rhythm rather than to the cortisol secretory burden.

## References

[1] Fleseriu M et al (2021) Consensus on diagnosis and management of Cushing’s disease: a guideline update, Dec. 01, Elsevier Ltd. 10.1016/S2213-8587(21)00235-7 10.1016/S2213-8587(21)00235-7 PMC8743006 34687601

[2] Gadelha M, Gatto F, Wildemberg LE, Fleseriu M Cushing’s syndrome. Dec 09 2023 Elsevier B V 10.1016/S0140-6736(23)01961-X 10.1016/S0140-6736(23)01961-X 37984386

[3] Ceccato F et al (2024) Clinical and biochemical data for the diagnosis of endogenous hypercortisolism: the ‘cushingomic’ approach. J Clin Endocrinol Metab Jul. 10.1210/clinem/dgae517 10.1210/clinem/dgae517 39056252

[4] Pivonello R, Isidori AM, De Martino MC, Newell-Price J, Biller BMK, Colao A (2016) Complications of Cushing’s syndrome: state of the Art. Lancet Publishing Group. 10.1016/S2213-8587(16)00086-3 10.1016/S2213-8587(16)00086-3 27177728

[5] Clayton RN et al (2016) Mortality in patients with Cushing’s disease more than 10 years after remission: A multicentre, multinational, retrospective cohort study. Lancet Diabetes Endocrinol 4(7):569–576. 10.1016/S2213-8587(16)30005-5 27265184 10.1016/S2213-8587(16)30005-5

[6] Pivonello R, De Leo M, Cozzolino A, Colao A (2015) The treatment of Cushing’s disease. Endocr Soc. 10.1210/er.2013-1048 10.1210/er.2013-1048 PMC4523083 26067718

[7] Petersenn S et al (2015) Outcomes in patients with Cushing’s disease undergoing transsphenoidal surgery: Systematic review assessing criteria used to define remission and recurrence. BioScientifica Ltd. 10.1530/EJE-14-0883 10.1530/EJE-14-0883 25599709

[8] Broersen LHA, Jha M, Biermasz NR, Pereira AM, Dekkers OM (2018) Effectiveness of medical treatment for Cushing’s syndrome: a systematic review and meta-analysis. Pituitary 21(6):631–641. 10.1007/s11102-018-0897-z 29855779 10.1007/s11102-018-0897-z PMC6244780

[9] von Elm E, Altman DG, Egger M, Pocock SJ, Gøtzsche PC, Vandenbroucke JP (2008) The Strengthening the Reporting of Observational Studies in Epidemiology (STROBE) statement: guidelines for reporting observational studies. J Clin Epidemiol 61(4): 344–349. 10.1016/j.jclinepi.2007.11.008 10.1016/j.jclinepi.2007.11.008 18313558

[10] Ceccato F (2024) The burden Of Cushing’s disease cardiometabolic comorbidities: comparison between surgical remission and long-term eucortisolism with medical treatment. Repository of the University of Padova. 10.25430/researchdata.cab.unipd.it.00001350

[11] Barbot M et al (2016) Second-line tests in the differential diagnosis of ACTH-dependent Cushing’s syndrome. Pituitary 19(5):488–495. 10.1007/s11102-016-0729-y 27236452 10.1007/s11102-016-0729-y

[12] ISO 15189:2012 Medical laboratories — Requirements for quality and competence. Published 2022. Accessed October 10 (2023) https://www.iso.org/standard/56115.html

[13] Ceccato F et al (2014) The diagnostic performance of urinary free cortisol is better than the cortisol: cortisone ratio in detecting de Novo Cushing’s syndrome: the use of a LC-MS/MS method in routine clinical practice. Eur J Endocrinol 171(1):1–7. 10.1530/EJE-14-0061 24743401 10.1530/EJE-14-0061

[14] Ceccato F et al (2012) Assessment of glucocorticoid therapy with salivary cortisol in secondary adrenal insufficiency. Eur J Endocrinol 167(6):769–776. 10.1530/EJE-12-0534 23034783 10.1530/EJE-12-0534

[15] Antonelli G, Ceccato F, Artusi C, Marinova M, Plebani M (2015) Salivary cortisol and cortisone by LC-MS/MS: Validation, reference intervals and diagnostic accuracy in Cushing’s syndrome. Clinica Chimica Acta 451:247–251. 10.1016/j.cca.2015.10.004 10.1016/j.cca.2015.10.004 26449783

[16] Ceccato F et al (2020) Dexamethasone measurement during low-dose suppression test for suspected hypercortisolism: threshold development with and validation. J Endocrinol Invest 43(8):1105–1113. 10.1007/s40618-020-01197-6 32060745 10.1007/s40618-020-01197-6

[17] Pivonello R, Faggiano A, Lombardi G, Colao A (2005) The metabolic syndrome and cardiovascular risk in Cushing’s syndrome. W.B. Saunders. 10.1016/j.ecl.2005.01.010 10.1016/j.ecl.2005.01.010 15850845

[18] Castinetti F et al (2014) Ketoconazole in Cushing’s disease: is it worth a try. J Clin Endocrinol Metab 99(5):1623–1630. 10.1210/jc.2013-3628 24471573 10.1210/jc.2013-3628

[19] Barbot M et al (2014) Combination therapy for Cushing’s disease: Effectiveness of two schedules of treatment. Should we start with cabergoline or ketoconazole? Pituitary 17(2):109–117. 10.1007/s11102-013-0475-3 23468128 10.1007/s11102-013-0475-3

[20] Colao A et al (1999) Persistence of Increased Cardiovascular Risk in Patients with Cushing’s Disease after Five Years of Successful Cure,., [Online]. Available: https://academic.oup.com/jcem/article/84/8/2664/2864186 10.1210/jcem.84.8.5896 10443657

[21] Ceccato F et al (2017) Sep., Body Composition is Different after Surgical or Pharmacological Remission of Cushing’s Syndrome: A Prospective DXA Study, Hormone and Metabolic Research, vol. 49, no. 9, pp. 660–666. 10.1055/s-0043-115008 10.1055/s-0043-115008 28718178

[22] Guarnotta V et al (2017) The degree of urinary hypercortisolism is not correlated with the severity of cushing’s syndrome. Endocrine 55(2):564–572. 10.1007/s12020-016-0914-9 26965912 10.1007/s12020-016-0914-9

[23] Giordano R et al (2011) Metabolic and cardiovascular outcomes in patients with Cushing’s syndrome of different aetiologies during active disease and 1 year after remission. Clin Endocrinol (Oxf) 75(3):354–360. 10.1111/j.1365-2265.2011.04055.x 21521323 10.1111/j.1365-2265.2011.04055.x

[24] Jha S, Sinaii N, McGlotten RN, Nieman LK (2020) Remission of hypertension after surgical cure of Cushing’s syndrome. Clin Endocrinol (Oxf), 92(20): 124–130. 10.1111/cen.14129 10.1111/cen.14129 PMC13502052 31721265

[25] Schernthaner-Reiter MH et al (2019) Factors predicting long-term comorbidities in patients with Cushing’s syndrome in remission. Endocrine 64(1):157–168. 10.1007/s12020-018-1819-6 30467627 10.1007/s12020-018-1819-6 PMC6453862

[26] Newell-Price J et al (2020) Use of late-night salivary cortisol to monitor response to medical treatment in Cushing’s disease. Eur J Endocrinol 182(2):207–217. 10.1530/EJE-19-0695 31804965 10.1530/EJE-19-0695 PMC7003692

[27] Mondin A et al (2023) Complications and mortality of Cushing’s disease: report on data collected over a 20-year period at a referral centre. Pituitary 26(5):551–560. 10.1007/s11102-023-01343-2 37495935 10.1007/s11102-023-01343-2 PMC10539191

[28] Barbot M et al (2018) Effects of pasireotide treatment on coagulative profile: a prospective study in patients with Cushing’s disease. Endocrine 62(1):207–214. 10.1007/s12020-018-1669-2 29980915 10.1007/s12020-018-1669-2

[29] Ferrante E et al (2022) Evaluation of procoagulant imbalance in Cushing’s syndrome after short- and long-term remission of disease. J Endocrinol Invest 45(1):9–16. 10.1007/s40618-021-01605-5 34115342 10.1007/s40618-021-01605-5 PMC8741706

[30] Zilio M, Barbot M, Ceccato F, Camozzi V, Bilora F, Casonato A, Frigo AC, Albiger N, Daidone V, Mazzai L, Mantero F, Scaroni C (2014) Diagnosis and complications of Cushing’s disease: gender-related differences. Clin Endocrinol (Oxf) 80(3):403–410. 10.1111/cen.12299 23889360 10.1111/cen.12299

[31] Detomas M, Deutschbein T, Tamburello M, Chifu I, Kimpel O, Sbiera S, Kroiss M, Fassnacht M, Altieri B (2024) Erythropoiesis in Cushing syndrome: sex-related and subtype-specific differences. Results from a monocentric study. J Endocrinol Invest 47(1):101–113. 10.1007/s40618-023-02128-x 37314685 10.1007/s40618-023-02128-x PMC10776705

[32] Fleseriu M et al (2019) Long-term efficacy and safety of once-monthly pasireotide in Cushing’s disease: A Phase III extension study. Clin Endocrinol (Oxf) 91(6):776–785. 10.1111/cen.14081 31465533 10.1111/cen.14081 PMC6899900

[33] Ceccato F et al (2018) Metyrapone treatment in Cushing’s syndrome: a real-life study. Endocrine 62(3):701–711. 10.1007/s12020-018-1675-4 30014438 10.1007/s12020-018-1675-4

[34] Minnetti M, Hasenmajer V, Pofi R, Venneri MA, Alexandraki KI, Isidori AM (2020) Fixing the broken clock in adrenal disorders: Focus on glucocorticoids and chronotherapy. BioScientifica Ltd. 10.1530/JOE-20-0066 10.1530/JOE-20-0066 32380472

